# Nerve Transplantation

**Published:** 1899-07

**Authors:** 


### Nerve Transplantation.

An interesting article in the *American Journal of the Medical Sciences*, reviews in a very thorough manner the subject of nerve transplantation, an operation often experimentally performed, but one that, as Dr. Peterson shows, has had as yet but a limited utilization in the human subject. While other methods, such as the Letievant flap or the insertion of catgut, may suffice in most cases where direct approximation is impracticable, there will be cases where, with proper facilities, nerve transplantation will be an advisable operation, and the microscopic examination of Huber tends to show that actual nerve tissue furnishes a better route for the down-growth of new axis-cylinders than any other foreign material. Nerve-flaps involve mutilation of healthy nerve and might, in case of very extensive removal of nerve-fiber, require dissection that would possibly have some inconvenience or danger. It is true that in the tabulated cases given by Dr. Peterson the success with very long implantations was poor, but the number of cases is yet too slight to justify any unfavorable conclusion as to the future possibilities with improved technic and experience. It is to be noted also in this connection that the case in which the most favorable results, almost a perfect cure, occurred, was that of Mayo Robson, where two inches of nerve were transplanted. In his earlier case, successful as far as regards restoration of sensation, some two and a half inches were resected. The old idea, still held by Kennedy, of direct reunion of the nerve or that of upward as well as downward growth of nerve-fibers, finds no support in these latest researches. The transplanted nerve simply serves as a convenient route for the regeneration from the central to the peripheral portion of the divided nerve, and the same purpose is served to a certain extent by the catgut bridge. It is not, however, altogether unreasonable to suppose that an identic tissue would have some advantage and might actually serve a purpose in the earlier restoration of function. The subject is one that has yet to be more studied before we can consider its surgical importance or even its physiologic bearings to be completely established. Dr. Peterson's article, besides being itself a valuable original contribution, adding to our knowledge of the subject, has the merit of presenting a very complete summary of the facts, so far as known, from all available sources.—*Journal Am. Med. Assn.*

